# Salivary leukocyte esterase activity by SillHa is a risk indicator of periodontal disease

**DOI:** 10.1186/s12903-023-02874-7

**Published:** 2023-03-30

**Authors:** Kyoko Ishii, Venkata Suresh Venkataiah, Takako Kajiwara, Kouta Umezawa, Shigeto Suzuki, Masato Nakano, Mayu Sawaguchi, Yoshio Yahata, Masahiro Saito

**Affiliations:** Division of Restorative Dentistry, Department of Ecological Dentistry, Tohoku University Graduate School of Dentistry, Sendai, Miyagi Japan

**Keywords:** Saliva test, Periodontal disease, Leukocyte esterase, Diagnostic marker, Oral hygiene

## Abstract

**Background:**

There is increasing evidence that diagnostic salivary tests measuring inflammatory biomarkers are being developed to assess inflammatory status for early detection, prevention, and progression of periodontal disease. Therefore, the aim of the present study was to investigate and identify the salivary biomarker that can predict the inflammatory status of periodontal disease.

**Methods:**

A total of 36 patients (28 women and 8 men) with an average age of 57 years were investigated. Unstimulated saliva was collected from the recruited subjects and analyzed using SillHa, a saliva-testing device that measures bacteria count, saliva buffer capacity, acidity, leukocyte esterase, protein, and ammonia. Periodontal parameters were then obtained by clinical examination and initial periodontal therapy was performed. Data obtained with SillHa were compared with clinical periodontal parameters at baseline, re-examination (three months from baseline), and final examination (six months from re-examination).

**Results:**

Leukocyte esterase activity in saliva measured by SillHa; BOP and PCR measured by clinical examination showed a significant difference between baseline and final examination and between re-examination and final examination. Patients in the lower median group (group 1) had a significant difference in leukocyte esterase activity between baseline and final examination and re-examination and final examination. In addition, patients in Group 1 had significantly lower BOP between baseline and final examination. While patients in the higher median group (group 2) showed a modest decrease in leukocyte esterase activity, which was significant only between baseline and final examination, no significant changes were observed concerning BOP. Furthermore, the associated systemic disease was observed in 30% and 81.2% of group 1 and 2 patients, respectively.

**Conclusion:**

The results suggest that leukocyte esterase activity in saliva measured by SillHa could serve as a reliable diagnostic marker for monitoring inflammatory status in periodontal disease.

## Introduction

Periodontitis is the most frequent immunoinflammatory disease in the dental field caused by infection with periodontal pathogens, leading to the destruction of periodontal tissue. Periodontal disease is often advanced when subjective symptoms occur at the time the patient is diagnosed; thus, periodontal treatment is often necessary, and sighted tooth loss in severe cases [1, 2]. Thus, risk assessment is important to prevent tooth loss due to periodontal disease [3]. Traditional diagnostic methods of periodontal disease by clinical examination to assess periodontal inflammation by analyzing plaque control records (PCR), probing pocket depth (PPD), bleeding on probing (BOP), and radiological examination useful in detecting existing periodontal disease status and are still routinely carried out in clinical practice [2, 4, 5]. Early detection of periodontal disease requires a diagnostic method that can detect the initial stage of inflammation, preventing further periodontal tissue destruction and planning effective periodontal therapy [6].

However, the risk of periodontal disease is related to many factors, including health status, host defense factors, and environmental factors, as well as individual differences [7]. Therefore, it is necessary to develop simple yet effective diagnostic techniques, such as identifying biomarkers to determine inflammatory status, so that easy to perform by patients on their own to understand the risk of periodontal disease and dentists can render effective periodontal therapy.

Gingivitis releases various inflammation-related factors, including leukocytes, lymphocytes and many others, which has led to the development of new medical equipment for the detection of inflammatory conditions, for example, identifying biomarkers to predict periodontal disease[8, 9]. A diagnostic testing technique using gingival crevicular fluid has been developed, and information regarding inflammatory response can be obtained. Still, the collection procedure is complicated and difficult for patients to perform by themselves. On the other hand, saliva testing is a simple method for analyzing periodontal inflammation because it can be collected quickly, noninvasively, and in addition, many inflammatory markers found in the blood can also be detected; thus, saliva is expected as an in vitro diagnostic tool for early diagnosis and prevention of periodontal disease [2, 4, 10–12]. Salivary tests used in dentistry include salivary volume, buffer capacity, bacterial culture, and acid-producing capacity and have been shown to be effective in changing patient behavior modification to prevent dental caries[13]. However, salivary tests for periodontal disease have been performed by analyzing R.N.A. and D.N.A. for specific inflammatory markers or by ELISA, but all these methods are single-task tests and require a long time before results are obtained [14–17]. Recent evidence has shown that microRNAs (miRNAs) are involved in several epigenetic processes associated with various diseases, including periodontitis [18–22]. MicroRNAs (miRNAs) are short, non-coding, single-stranded RNA molecules that have been associated with the release of inflammatory cytokines and metalloproteases in gingival fibroblasts in the early stages of periodontitis, suggesting that miRNAs may play an important role in the early stages and later progression of the disease [20]. miRNAs present in various biofluids such as blood, serum, GCF, and saliva, providing high availability and the opportunity to use as a diagnostic marker for several systemic and oral inflammatory diseases [18, 21, 23]. Numerous studies on miRNAs have been conducted to identify miRNAs as reliable biomarkers for the diagnosis of various diseases, including periodontitis. However, their detectability and expression in saliva as non-invasive markers are questionable due to limited clinical data. Therefore, it is difficult to use them routinely in clinical practice. This underlies the need for a more straightforward diagnostic technique to prevent or identify periodontal disease risk assessment.

In a recent study, a urinary test strip was used to identify the salivary biomarkers to diagnose the risk and severity of the periodontal disease. The study’s results showed that when the test strip is moistened with saliva from patients with severe periodontitis, high levels of lactoferrin, hemoglobin, and leukocytes, indicate that periodontal disease can be tested noninvasively [4]. Similar to this technology, a saliva-testing device: SillHa, has been developed that uses a two-wavelength reflectance measurement method and then uses a proprietary algorithm to analyze multiple parameters, including bacteria count, saliva buffer capacity, acidity, leukocyte count, protein amount, and ammonia content and calculate quantitative values in a short period [24].

In addition, PPD and BOP tests of mild, moderate, and severe periodontal disease showed a significant correlation with the results of occult blood, leukocyte count and protein amount indicating the validity and reliability of periodontal disease-related measurements by the SillHa. These results suggest that the measurement of inflammatory markers by saliva testing helps understand the inflammatory status of periodontal disease but may also help improve patient motivation toward treatment and self-care at the dental clinic, taking advantage of the short time required for a multiparameter test. There are few reports evaluating the validity and reliability of saliva testing instruments, but their usefulness in actual practice is unknown.

Based on these findings, the aim of this study is to investigate the correlation between the items measured by the saliva testing device and the periodontal examination parameters during periodontal treatment at baseline, re-examination, and final examination, to validate the usefulness of the saliva diagnostic method. In addition, the secondary objective is to investigate the correlation of the saliva testing device with those of the conventional saliva test and periodontal risk systems such as the cariogram and OHIS.

## Materials and Methods

### Patients

In accordance with the Declaration of Helsinki, this study was approved by the Ethics Committee of Tohoku University School of Dentistry (No. 2018-3-025). This study was designed as a single-center prospective cohort study. Inclusion criteria include patients at least 20 years of age with periodontal disease, including gingivitis, periodontitis, attachment loss, or bone resorption at stage II, grade A or higher on periodontal examination, and with at least eight years since the onset of periodontal disease. All the teeth subjected to periodontal examination are all teeth except wisdom teeth. Wisdom teeth are not included because pericoronitis and tooth impaction may cause isolated periodontal defects that may negatively influence the outcome of the study. Exclusion criteria included smokers, patients with systemic diseases and mental illness, pregnant women and individuals who were undergoing periodontal treatment at the time of the study, as this may negatively affect the results of the study. We also investigated the systemic disease associated with the participants. Patients were given a detailed study description, including a brochure explaining the study design, and signed an informed consent form. The study was conducted from November 2018 to March 2020.

## Periodontal examinations

**Fig. 1:**
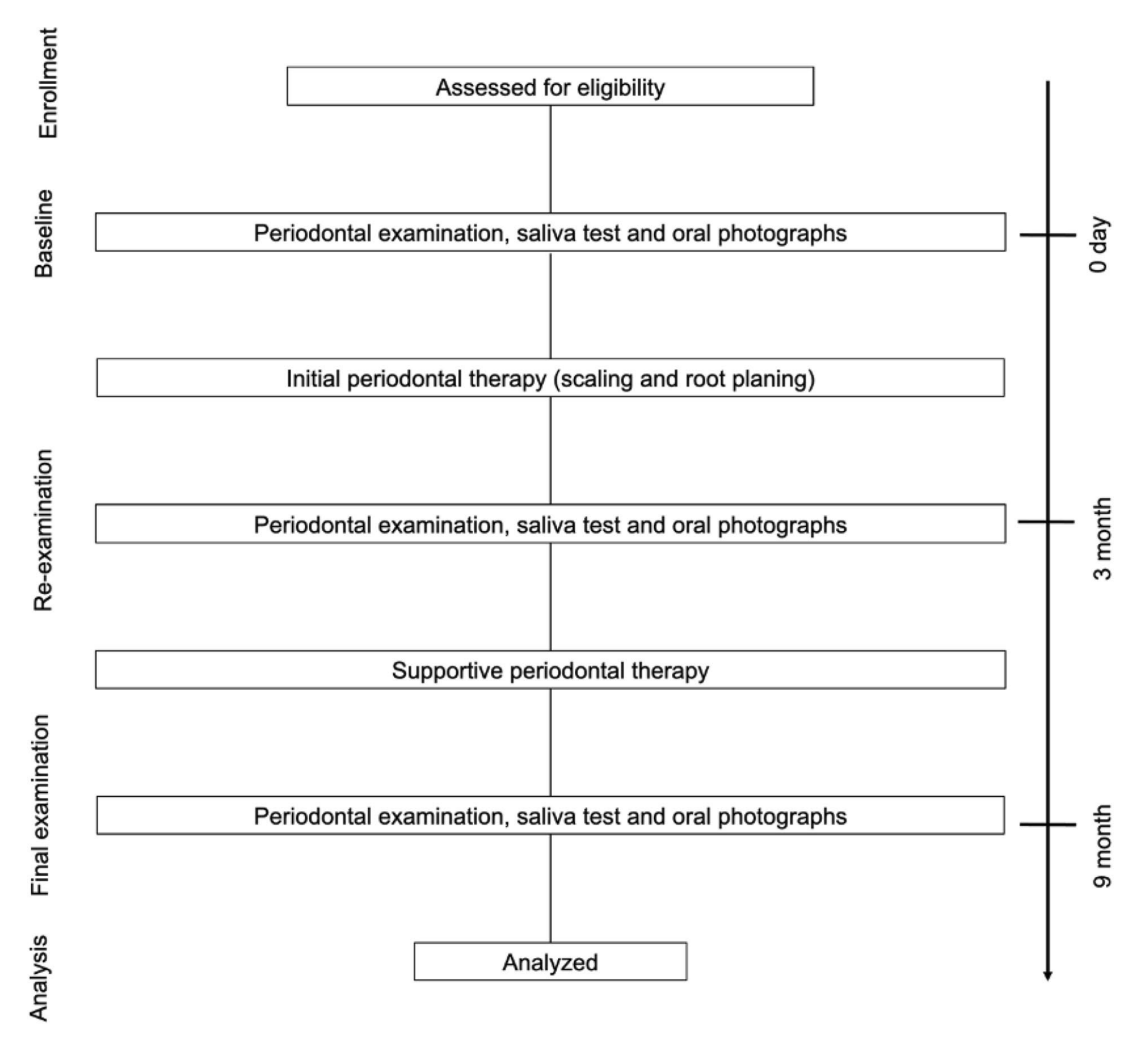
**Study design.** At the baseline examination, all the study subjects underwent an initial periodontal treatment, including scaling and root planing. Re-examination by supportive periodontal therapy was carried out three months after the baseline examination. Final examination were carried out six months after the re-examination. All the study participants underwent periodontal examinations via saliva tests, and oral photographs were taken during the baseline examination, re-examination, and final examination period.

At the baseline for all patients, a periodontal examination was performed to assess probing pocket depth (PPD) and bleeding on probing (BOP) for 6 surfaces for all teeth and initial periodontal therapy (scaling and root planning) was carried out. The periodontal examination was performed by a qualified dental hygienist who was trained to apply pressure force using calibrated periodontal probes with a pressure calibration of 20 g/0.2 N with models. Intraoral photographs were taken for all patients at the baseline examination, re-examination (three months after the baseline examination) and final examination period (six months after reevaluation) to document visual gingival changes. All the study subjects visited clinics every three months for supportive periodontal therapy (SPT) after reevaluation, and these treatments continued as maintenance therapy (Fig. 1) until final examination period. Adequate oral hygiene instructions were given to all the study participants during the initial periodontal treatment and SPT.

## Saliva examinations

The caries risk test kit “Dentocult” (Orion Diagnostica Oy, P.O. Box 83, FI-02101 Espoo, Finland) was used for all patients following the method specified by the vendor to determine salivary buffer capacity, bacterial content (Streptococcus mutans, Lactobacillus bacteria) and saliva volume. The levels of S. mutans and lactobacilli were evaluated according to the manufacturer’s chart: class 0: <10.000 CFU/ml, class 1: <100.000 CFU/ ml, class 2: 100.000–1.000.000 CFU/ml, class 3: > 1.000.000 CFU/ml [25].

## OHIS examination

For periodontal disease risk testing, OHIS (Oral Health Information Suite) was performed using PreViser (New Hampshire, U.S.A.: Concord NH, URL. https://www.previser.com). The following examination items were entered into the OHIS software: name, gender, date of birth, frequency of visits, smoking history, presence of systemic disease, oral status after the examination such as plaque control, probing depth, bleeding on probing, distance from C.E.J on radiographs, and accurate clinical data of the patient. The risk of periodontal disease (1–5) and disease status (1-100) are then quantified and analyzed as risk scores and disease scores from the vast amount of epidemiological data and evaluated graphically.

## Saliva examination using SillHa

The sample collection for the saliva test using SillHa was performed according to manufacturing instructions (https://www.arkrayusa.com/sites/arkrayusa.com/files/SiLL-Ha_Operating_Manual.pdf) and previous report [24]. The patients were asked not to eat, drink, or clean their mouths for at least 2 h before the examination by SillHa. All participants were asked to spit into a sterile paper cup after swishing with 3 ml of distilled water for 10 s. A droplet of saliva was collected using a dropper, placed on an analyte pad of the SillHa paper test strip, and loaded into the instrument for automated measurement. The SillHa salivary analyzer data measurement ranges between 0 and 100 correlate with the following scores that were established by the device manufacturer, ARKRAY: cariogenic bacteria (10^6^-10^8^Colony Forming Units (CFU)/mL); acidity (pH 6.0–8.0); buffer capacity (pH 2.8-6.0); blood (0-0.50 mg/dL); leukocyte (0-200 U/L); protein (0–60 mg/dL); and ammonia (0–10,000 N-µg/dL) [24].

## Statistical analysis

The sample size was calculated based on similar research, which showed that the correlation coefficient, r = 0.2–0.6 between the salivary test and periodontal examination [26]. The sample size was determined to estimate the correlation coefficient at 0.45 with α error of 0.05 and a power of 0.80. The minimum sample size was calculated to be 36. After taking dropouts into account, we decided to recruit 40 cases. First, we evaluated the normality of the data obtained using the Shapiro-Wilk test. Since most data departed from the normal distribution, a nonparametric Spearman’s rank correlation was calculated to evaluate the correlation coefficient between saliva tests using SillHA and periodontal examinations. A nonparametric Friedman test was performed to compare three time points (baseline, re-examination, and final examination) of the saliva test and clinical parameters. For the comparison between groups 1 and 2, the difference in the leukocyte esterase activity and BOP at the final examination was compared using an analysis of covariance (ANCOVA) performed with age, sex, BOP as an indicator of periodontal status at baseline, and smoking status as a confounding factor. Then, a two-way repeated-measures analysis (ANOVA) was used to examine whether there was a difference in leukocyte esterase activity and BOP between time points and groups and the interaction between them. Tukey post hoc test was performed to detect significant differences between groups. All statistical analyses were performed with GraphPad Prism 9 (GraphPad Software, San Diego, CA, U.S.A.) and IBM SPSS Statistics V21.0 (IBM coro., Chicago, IL). The significance level was set at alpha = 0.05.

## Results

### Study participants

We recruited 40 participants at the baseline, and a total of 36 patients (28 women and 8 men) with a mean age of 57 years were investigated at the final examination. The detailed information of the participants is described in Table 1.

**Table 1:** Characteristics of participants at the time of baseline examination.

Periodontal examination							
Average TN	Average PPD	Average BOP	Average PCR		Smoking		
25.8 (0.6) *	2.9 mm (1.2) *	13.5% (16.5) *	37.7% (15) *		2.78%		
Systemic diseases							
Diabetes	Cardiovascular disease	Autoimmune disease	Bone disease	Inflammatory disease		Mental disorder	
5.56%	33.33%	27.8%	2.78%	2.78%		2.78%	

TN, number of teeth present; PPD, probing pocket depth; BOP, bleeding on probing; PCR, Plaque control record; SD, standard deviation. *Mean (SD).

## Caries risk assessment

**Fig. 2:**
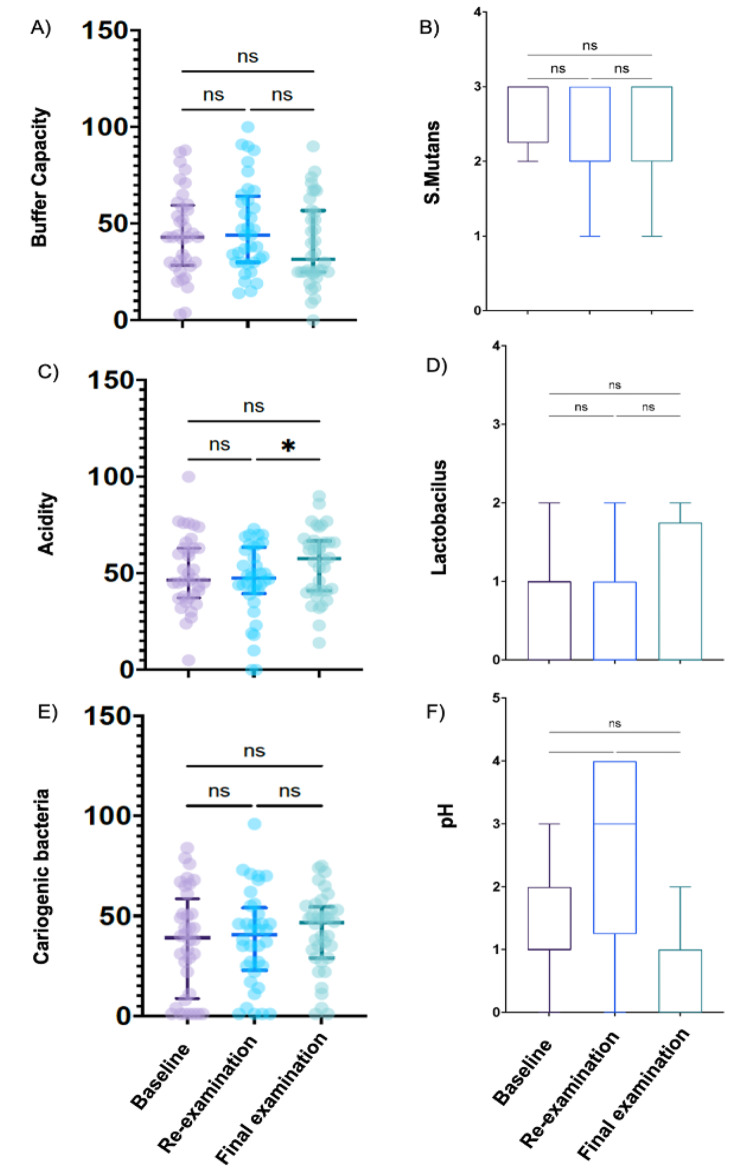
Comparison of the saliva test using bacterial culture, litmus paper (pH) and SillHa examination. Comparison of test paper for analyzing buffer capacity (A), acidity (C), cariogenic bacteria (E) and bacterial quantitative analysis including S.Mutans (B), Lactobacillus (C) or litmus paper for analyzing pH (F) at the baseline, re-examination, and final examination. *P < 0.05. NS: Not significant.

To analyze the risk of dental caries by Dentocult, we analyzed the bacterial content and buffering capacity of S.mutans and Lactobacillus, which are representative caries-causing bacteria. The results showed no significant difference in bacterial quantification and their buffering capacity at the baseline, re-examination, and final examination saliva tests (Fig. 2B, D and F).

Similarly, there were no significant differences in caries-related parameters such as caries count, acidity, and buffering capacity between the baseline, re-examination, and final examination, indicating that these parameters were not affected by oral care (Fig. 2A, C and E).

## Comparison of clinical periodontal examination and SillHa examination

**Fig. 3:**
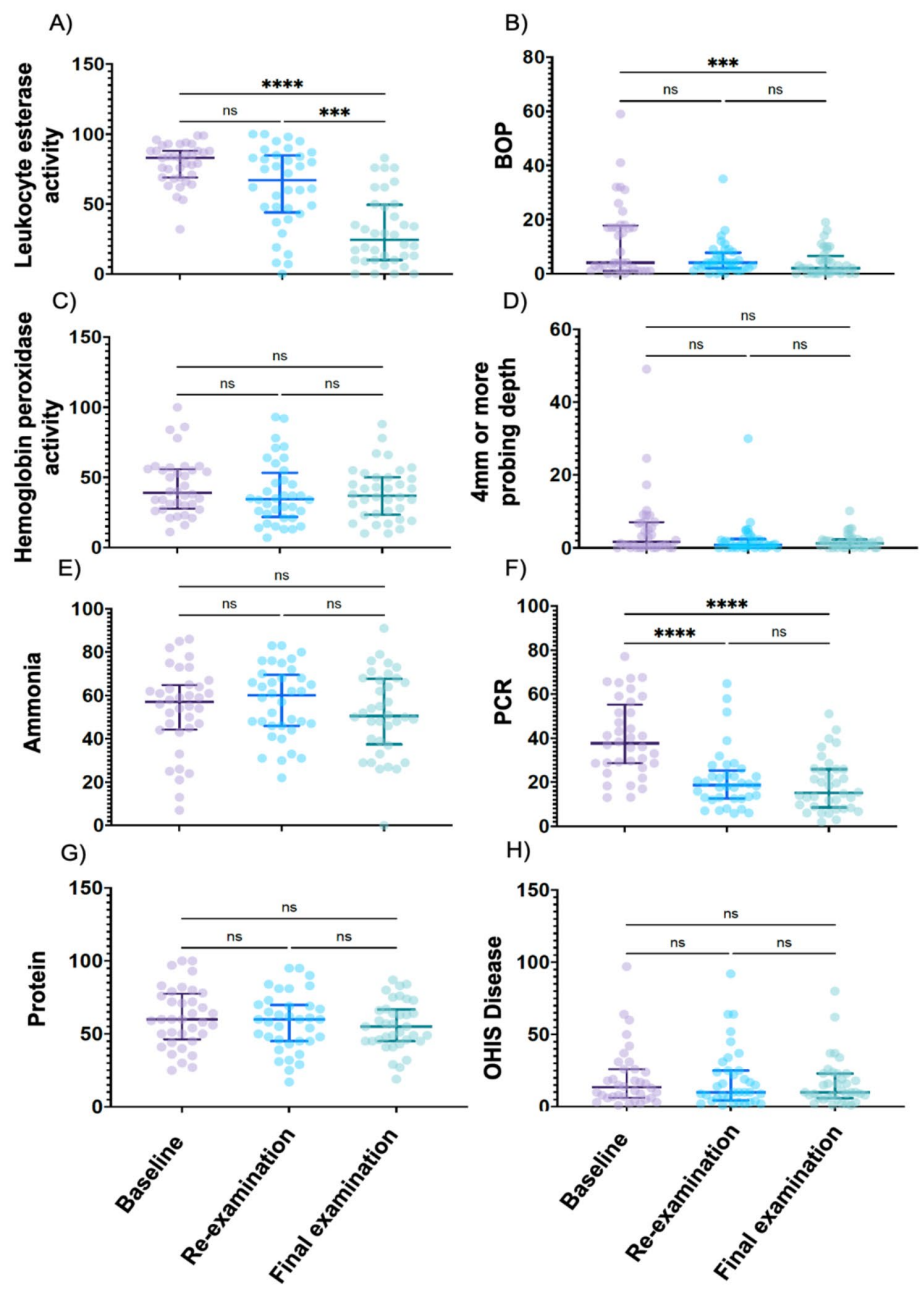
Comparison of the SillHa examination and periodontal examination. Comparison of test paper for analyzing Leukocyte esterase activity (A), hemoglobin peroxidase activity (C), ammonia (E) or protein (G) at the baseline, re-examination and final examination. Comparison of periodontal examination parameters, including BOP (B), 4 mm or more probing depth (D), plaque control record (PCR) (F) or OHIS disease rate (H) at the baseline, re-examination, and final examination. *P < 0.05. ***P < 0.01. ****P < 0.0001. NS: Not significant.

The correlation coefficients between the salivary test and periodontal examination in this study ranged from 0.04 to 0.37, which was comparable to previous studies [26]. On the other hand, significant differences in BOP and PCR were found between the baseline and final examination, but not between the baseline and re-examination, nor between re-examination and final examination although no significant difference was observed in periodontal pockets larger than 4 mm (Fig. 3B, D, F and H). In addition, the OHIS, which validates the risk test for periodontal disease, showed no significant difference between the baseline, re-examination, and final examination. The results showed no statistically significant differences in the periodontal pocket examination (> 4 mm) and OHIS state because there were no patients with severe periodontal disease. However, the significant differences in BOP and PCR indicate that the treatment of periodontal disease was effective.

Next, the analysis of inflammatory parameters measured using SillHa showed no significant difference in leukocyte esterase activity between the baseline and re-examination but demonstrated a significant difference between the baseline and final examination and between re-examination and final examination. On the other hand, there were no significant differences in hemoglobin peroxidase, ammonia analysis and protein content (Fig. 3A, C, E and G). These results indicate that the effectiveness of periodontal treatment may be correlated with the amount of leukocyte esterase activity in saliva.

**Fig. 4:**
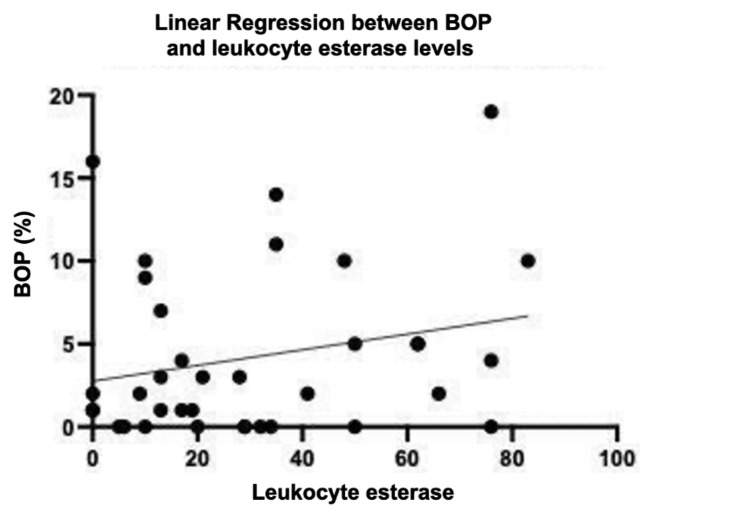
Correlation of leukocyte esterase and BOP. Comparison of test paper for analyzing Leukocyte esterase activity and BOP at the baseline and final examination. r^2^ = 0.037.

However, a comparison of leukocyte esterase levels at baseline and final examination revealed a low correlation, suggesting that levels did not decrease uniformly in response to treatment. Based on this result, we hypothesized that the patients whose esterase did not change with periodontal treatment were treatment-resistant groups (Fig. 4). To implement this hypothesis, we divided the population with lower leukocyte esterase activity than the median value into a group with improved gingival inflammation (group 1) and a group with higher than median into high-risk populations (group 2). The results of the ANCOVA, examining the effect of confounding between each group, revealed an interaction between gender (p = 0.0031) and BOP (p = 0.0442), and the significance of the regression for age (p = 0.2644) and smoking (p = 0.3973) was rejected. Two-way ANOVA showed an interaction in esterase activity between groups and examination time-point, but no interaction was observed in BOP.

**Fig. 5:**
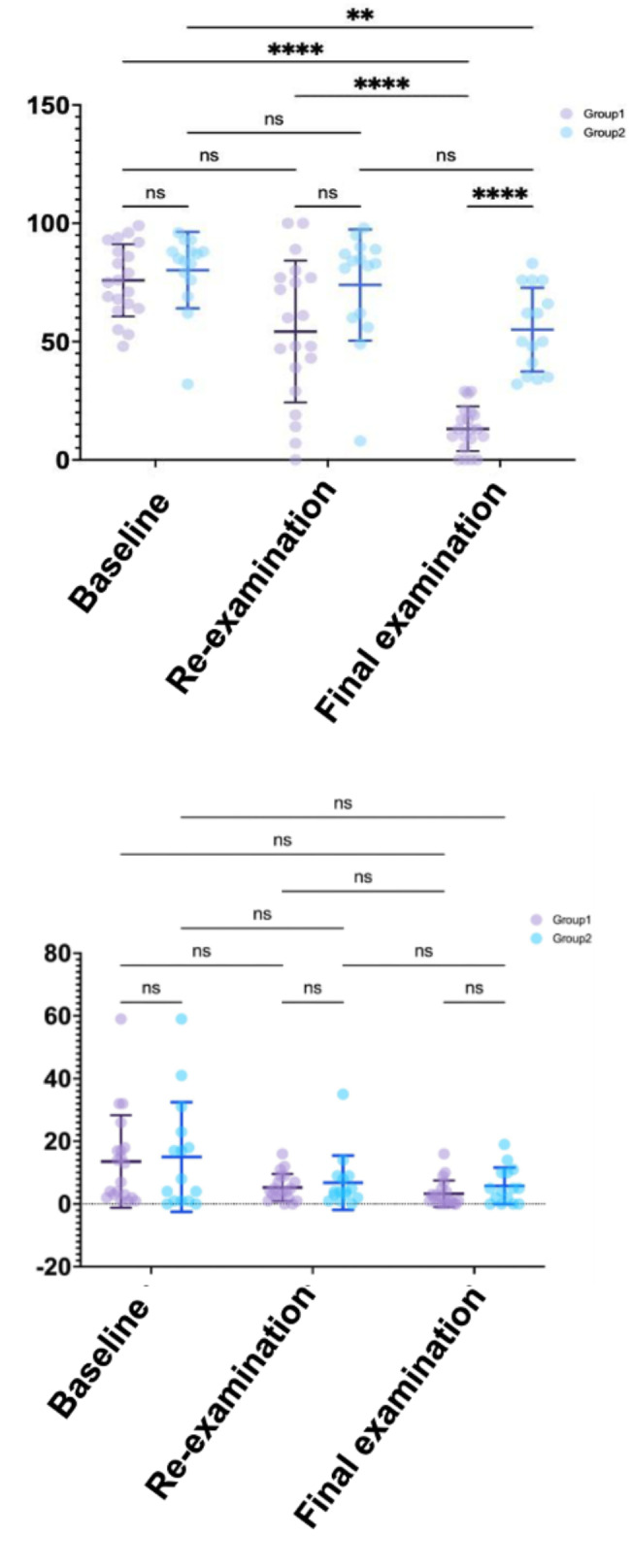
Comparison of leukocyte esterase activity and BOP in the patients below (group 1) or above (group 2) median groups. Comparison of leukocyte esterase activity (upper panel) or BOP (lower panel) in group 1 (purple circle) or group 2 (blue circle) at the baseline, re-examination, and final examination. *P < 0.05. ***P < 0.01. ****P < 0.0001. NS: Not significant.

For all the study subjects, the results of group 1 clearly showed decreased leukocyte esterase activity from the baseline, until the final examination, with statistically significant differences between the baseline and final examination and between re-examination and final examination (Fig. 5 upper panel). In contrast, a modest improvement in leukocyte esterase activity was observed in Group 2 from the baseline until the final examination compared to group 1 with statistically significant differences only at the baseline and final examination (Fig. 5 upper panel). For BOP, significant improvement was observed between the baseline and final examination in both Group 1 and Group 2 patients (Fig. 5 lower panel).

## Relevance of systemic disease and leukocyte esterase activity

The results indicated that the risk of periodontal disease could be distinguished by the activity of leukocyte esterase in saliva. We then analyzed the presence of systemic diseases associated with the risk of periodontal disease. The results showed that 30% of Group 1 patients had cardiovascular disease (hypertension), psychiatric disease, diabetes, while 81.2% of Group 2 patients had cardiovascular disease (hypertension, valvular disease, bradycardia), diabetes, osteoporosis, renal disease, and irritable bowel syndrome, an inflammatory disease (Table 2). These results indicate that leukocyte esterase may be a salivary biomarker reflecting periodontal disease status and risk by saliva testing.

**Table 2:** Comparison of systemic disease prevalence rate and leukocyte esterase activity in the patients with below (Group 1) or above (Group 2) median groups

	Leukocyte esterase activity	
	Group 1	Group 2
Number of patients	20	16
Prevalence (%)	30	81.2
Cardiovascular disease	4	8
Mental disorder	1	0
Autoimmune disease	0	1
Bone disease	0	1
Renal disease	0	1
Diabetes	1	1
Inflammatory disease	0	1

## Discussion

In the present study, although there was no significant difference in the caries-related parameters, we found that measurement of leukocyte esterase, a factor related to periodontal disease, is a risk indicator for periodontal disease and can be performed by patients at home during the periodontal maintenance phase. We also found that patients who had a higher leukocyte esterase group at the final examination had a higher risk of periodontal disease associated with systemic diseases such as diabetes, cardiovascular, renal, or autoimmune diseases. The diagnosis of periodontal disease is mainly performed by clinical evaluation of periodontal tissues and supported by radiographs. After confirming the disease status, it is possible to provide appropriate guidance and regular plaque control measures to maintain dental and oral health through periodic oral maintenance. In this study, periodontal disease treatment reduced BOP, PCR, and 4 mm pocket depths or greater following initial periodontal therapy, re-examination, and final examination. However, the disadvantage of this method is that it requires a visit to the dentist’s office to evaluate periodontal disease status and cannot be performed by the patient at home. To assess the usefulness of saliva testing using absorbent paper in periodontal disease, subjects were monitored during periodontal treatment, including baseline, re-examination and final examination, a long-term observation after one year at home during the periodontal maintenance phase.

Clinical evaluation of periodontal disease is investigated by clinical and radiographical examination to detect the periodontal tissue destruction level [27]. However, conventional diagnostic methods are not very effective in determining the active site of disease activity and predicting future tissue destruction [28, 29]. Therefore, a paradigm shift towards understanding the pathogenesis of periodontal disease for prevention and treatment requires objectivity in diagnostic methods, including sensitivity and specificity, as well as an explanation of disease intensity. In addition, the recent COVID-19 pandemic has reduced patients’ visits to the dentist [30]. A remote diagnostic system that can be easily performed by patients themselves and quickly determine periodontal disease status is much needed. Salivary biomarkers are relatively easy to obtain noninvasively and are beneficial for diagnosing periodontal disease [28]. Biomarkers allow the monitoring of disease-related biochemical processes and provide insight into individual cases. The expression of proinflammatory markers such as TNF-α, IL-6, IL-8, IL-17 [31], and IL-1β [5, 32] has been reported to be detected in the gingival crevicular fluid after gingivitis and can also be detected in saliva. Metalloproteinase (M.M.P.)-8, another inflammatory marker, has recently been reported to be a biomarker for in vitro diagnosis of severe periodontal disease. Evidence from other studies suggests that microRNAs (miRNAs) are being extensively studied to validate their use as potential biomarkers for the diagnosis of periodontal disease [20, 21, 33]. A recent study reported that miRNA in GCF (gingival crevicular fluid) was different in healthy subjects compared to periodontitis and in periodontitis-positive CVD patients [18]. In another study, Fujimori et al. demonstrated an association between salivary miRNA levels and periodontitis progression in a two-year cohort study [21]. These data suggested that miRNA could serve as novel diagnostic markers for periodontitis. In addition, tacrolimus, an immunosuppressive drug ointment, showed effective improvement in the signs and symptoms of oral lichen planus compared with the anti-inflammatory mouth rinse at the 3-month follow-up, suggesting that biomarkers related to immune response may be a useful indicator for early detection of periodontal disease [34]. Similarly, in this study, we demonstrated the possibility of determining periodontal disease risk by measuring leukocyte esterase activity over time in saliva. We also found that even after the initial treatment of periodontal disease, at final examination, the population in the higher median group had an increased proportion of patients with systemic diseases, including cardiovascular disease, inflammatory disease, and osteoporosis, and was a high-risk population for periodontal disease. These findings suggested that leukocyte esterase could be a biomarker for the prediction of periodontal disease risk.

Neutrophils are produced in the bone marrow and circulate in capillaries under normal conditions but are released outside of blood vessels in response to inflammatory cytokines produced during inflammatory responses by periodontal pathogens and are thought to play a role in the removal of infected material [35], Leukocytes are mobilized mainly to the gingival sulcus by periodontal disease, a process that is triggered by a bacterial infection in the gingiva, in which endothelial cells of surrounding capillaries express selectins to induce rolling, followed by integrins to promote firm adhesion and migration out of the vessel, phagocytosis, generation of reactive oxygen species, the release of granular material, production of cytokines and formation of neutrophil extracellular traps, which facilitate microbial clearance through a variety of processes [36, 37]. Leukocytes have long been thought to play a role in the early stages of periodontitis or acute inflammation, but recently it has been reported that they also play an important role in chronic inflammation [38]. Neutrophils in the oral tissues of patients with chronic periodontitis have been shown to have a longer life span than neutrophils in the oral tissues of healthy subjects and have been reported to contribute not only to connective tissue damage but also to sustained bone resorption via the release of esterase and M.M.P. s [19]. In this study, comparison of laboratory values and saliva tests during periodontitis treatment showed that leukocyte esterase did not decrease upon re-examination, which was associated with a decrease in BOP. This result indicates that differentiated neutrophils are mobilized even though capillary dilation has improved as determined visually by bleeding and that the activity of these neutrophils may decrease after the initial treatment is completed and SPT is continued. In addition, patients with high leukocyte esterase levels at final examination after SPT were predicted to be at increased risk for the periodontal disease since neutrophils are easily mobilized to the periodontal tissues as a complication of systemic disease. Therefore, it is strongly suggested that patients with high leukocyte esterase levels require frequent periodontal disease treatment and maintenance therapy.

The leukocyte esterase test using absorbent paper was developed to recognize the presence of leukocytes in urinary tract infections. Since it is inexpensive and can be performed at room temperature, this system is used not only for urinary tract infections but also for diagnosing meningitis, peritonitis, abdominal trauma or helicobacter infection [4]. Among these, combinations of synovial leukocyte esterase activity measured by urine dipstick and white blood cell counting were used to diagnose arthritis and determine inflammation in the orthopedic surgery setting [39, 40]. Regarding the relationship with periodontal disease, a previous report indicated that quantitative analysis of leukocytes is associated with the severity of periodontal disease since leukocyte-related proteins, LFA-1 and ICAM-1, are elevated in unstimulated and stimulated saliva of stage III and grade B periodontitis patients compared to healthy controls. [4].

Like these results, our data show that leukocyte esterase is a valuable parameter for the high-risk population for periodontal disease. Thus, leukocyte esterase activity in saliva could determine the extent of periodontal disease activity. In the present study, supportive periodontal therapy (SPT) was performed between the baseline and re-examination Therefore, the period after completion of the initial treatment is not uniform among the study participants. Hence, the results of the periodontal examination and SillHa measurement parameters, including leukocyte esterase, may not be equally comparable among all study participants at the time of re-examination. This could be a potential limitation of the study and will be addressed in future research by establishing a standard study design. Although further analysis is required, a saliva test with leukocyte esterase activity could serve as a remote diagnostic tool for patients requiring periodontal treatment.

## Conclusion

The results of this study suggest that a biochemical assay measuring leukocyte esterase in saliva could be a diagnostic biomarker to predict the inflammatory status of periodontal disease with relative ease compared to conventional periodontal tests. However, further clinical studies are needed to validate remote periodontal disease risk assessment using salivary leukocyte esterase as an indicator.

## Data Availability

The datasets generated and analyzed during the current study are available in the figshare repository: https://figshare.com/s/ba76e893aba9bae4ff7a.
